# Exploring the psychogastroenterology patterns of functional dyspepsia and irritable bowel syndrome comorbidities based on latent profile analysis

**DOI:** 10.3389/fphar.2026.1759170

**Published:** 2026-09-16

**Authors:** Zhongyu Huang, Zipan Lyu, Fengbin Liu

**Affiliations:** 1 Department of Clinical Nutrition, Guangzhou First People’s Hospital School of Medicine, South China University of Technology, Guangzhou, Guangdong, China; 2 The First Affiliated Hospital of Guangzhou University of Chinese Medicine, Guangzhou, Guangdong, China; 3 Integrated Chinese and Western Medicine Postdoctoral Research Station, Jinan University, Guangzhou, China; 4 School of Biological Sciences, Nanyang Technological University, Singapore, Singapore

**Keywords:** functional dyspepsia, heterogeneity, irritable bowel syndrome, latent profile modeling, multidimensional computerized adaptive testing

## Abstract

**Background:**

Comorbidities between different subtypes of functional gastrointestinal disorders (FGIDs) are highly prevalent in clinical practice. The heterogeneity of clinical appearance of these comorbidities causes difficulties with individualized diagnosis and comprehensive treatment of FGIDs. In this study, we aimed to estimate and interpret the psychology–gastroenterology interaction patterns of FGIDs for heterogeneous clinical appearances against the traditional anatomic diagnosis criteria.

**Methods:**

In a retrospective cross-sectional study, the intensities of clinical appearances covering gastrointestinal symptoms, systemic symptoms, psychological disorders, and environmental sensitivity were collected using a self-administered questionnaire. Then, a multidimensional assessment framework was applied to estimate the quantitative model using structural equation modeling (SEM) and item response theory (IRT). The latent traits of each patient were evaluated using multidimensional computerized adaptive testing (MCAT). Lastly, the clinical patterns were estimated using either the original symptom intensity or quantified trait scores to interpret the psychology–gastroenterology interaction characteristics of FGIDs.

**Results:**

A total of 996 patients diagnosed with functional dyspepsia (FD) or irritable bowel syndrome (IBS) were enrolled in the study; the comorbidities of these two subtypes were observed along with the heterogeneities of their clinical appearances. The multidimensional framework was evaluated to be adequate based on the following parameters for SEM: Chi-square/df = 3.45, comparative fit index (CFI) = 0.92, goodness-of-fit index (GFI) = 0.96, root mean-squared error of approximation (RMSEA) = 0.05, Tucker–Lewis index (TLI) = 0.90, and root mean-squared residual (RMR) = 0.02. Based on the 5-dimension latent trait scores, the 7-latent-profile model was evaluated to be adequate for representing the heterogeneity of patients with comorbidities between FD and IBS (entropy = 0.98, Lo–Mendell–Rubin likelihood ratio test (LMRT) *p*-value < 0.01, bootstrap likelihood ratio test (BLRT)-*p*-value < 0.01). The model also represented different patterns of psychology–gastroenterology interactions for the heterogeneous clinical appearances.

**Conclusion:**

Through the application of multidimensional variable analysis, the paradigm proposed in this study equips clinicians with an approach for quantifying and interpreting the complex clinical patterns of FGIDs.

## 1 Introduction

Functional gastrointestinal disorders (FGIDs) are known to affect over 40% of the population worldwide (Black et al., 2020; Sperber et al., 2021). Although symptom-based criteria, such as the Rome IV criteria, serve as the guidelines for categorizing FGIDs in research design and clinical practice, the heterogeneous and variable clinical manifestations of FGIDs make it difficult for clinicians to arrive at individualized diagnoses and further hinder satisfactory treatment efficacy without obvious organic pathologies (Lin and Chang, 2018). The prevalence of comorbidities among FGID subtypes is reportedly high, especially those between functional dyspepsia (FD) and irritable bowel syndrome (IBS) (Nakov et al., 2020; Park, 2011; Von Wulffen et al., 2019). FD and IBS are common pathophysiological disturbances that reportedly involve gastrointestinal (GI) motility dysfunction (Carbone et al., 2020; Tanaka et al., 2018), visceral hypersensitivity (Simrén et al., 2018), immune activation (Burns et al., 2019), abnormal brain–gut interactions (Drossman and Hasler, 2016), and psychosocial factors (Levy et al., 2006). Thus, the interactions among psychological and gastroenterological disorders should be addressed for promoting the individual wellbeing of patients with FGIDs (Fikree and Byrne, 2021). These observations evidence the importance of comprehensive assessments in clinical practice (Gwee and Chua, 2006). However, there are obvious gaps between the scheduled anatomic symptom-based rules of the Rome criteria and individualized evaluations of the heterogeneous clinical appearances of FGIDs (Spiller et al., 2010). Hence, the rules for subgrouping FGIDs should be more comprehensive and precise (Siah et al., 2018).

Various instruments have been developed in recent times to assist with the clinical assessments of FGIDs (Gierk et al., 2014; Kanazawa et al., 2015). Interdisciplinary methods have also been applied to explore hidden patterns in the heterogeneous clinical appearances of diseases (Bouchoucha et al., 2020). For example, principal component analysis and latent class analysis (LCA) were used to identify subgroups of FGIDs by clustering symptoms and extracting relationships from the complex clinical manifestations (Siah et al., 2018; Polster et al., 2019; Polster et al., 2017). As advanced applications, informatics platforms such as the Rome IV multidimensional clinical profile and Interactive Clinical Decision Toolkit have also been developed to support individualized management of FGIDs (Chey et al., 2012; Drossman et al., 2009; Drossman, 2016). However, cultural differences have necessitated adjustment of these instruments to meet the needs of regional practice. Moreover, interpretation of these instruments should be optimized to support decoupling interactions among the latent traits of patients with FGIDs in clinical practice.

Innovative testing theories and algorithms, such as multidimensional item response theory (MIRT) and structural equation modeling (SEM), have been applied to enhance the feasibility and interpretability of assessments in specific occasions (Huang et al., 2020; Dowling et al., 2016). In these paradigms, the conceptual framework could be constructed and validated via SEM while the psychometric properties of the instrument could be estimated with MIRT. In a flexible framework, computerized adaptive testing (CAT) on the basis of MIRT was also shown to enable adaptive quantification of the latent traits of the examinees (Goldenberg et al., 2018; Chalmers, 2016). Therefore, the present study was aimed at exploring the hidden patterns in the heterogeneous clinical appearances of patients with comorbidities between FD and IBS by taking advantage of these multidisciplinary approaches.

## 2 Materials and methods

### 2.1 Self-administered questionnaire and assessment framework

Following a comprehensive review on the clinical prevalence of FGID-related symptoms, we constructed an item pool by referring to the consensus of five regional experts and clinical guidance on FD and IBS published in Asia (Miwa et al., 2012; Gwee et al., 2010; Hu, 2008; Li et al., 2017; Li et al., 2018). The items were designed by referring to the settings of scales, including IBS-SSS (Francis et al., 1997) and SF-36 (Li et al., 2003). Literature adjustment was then carried out through a group discussion among clinical practitioners and experts. Symptoms such as “abdominal cold” (a subjective sensation of coldness in the abdomen often accompanied by pain or discomfort, which is typically relieved by applying warmth) and “overwork aggravated discomfort” (discomfort triggered or worsened by physical or mental exhaustion that tends to improve with rest) that are not specified in the Rome criteria were discussed and added to the assessment upon considering the regional clinical prevalence and cultural norms. The categorized options were designed to assess the intensities of IBS- and FD-related symptoms. In terms of the influencing factors, including dietary, environmental, and mental disorders, dichotomous items were designed for inductively discriminating and alleviating factors associated with clinical discomfort. Finally, the designed questionnaire consisted of 26 items in the assessment framework and covered five latent traits, including GI symptoms, systemic symptoms, environmental sensitivity, and emotional discomfort.

### 2.2 Participants

A cross-sectional study was designed for collecting the clinical features of patients diagnosed with FD and IBS according to the Rome IV criteria. Patients with organic disease and those with severe systemic disease or with a history of drug or alcohol abuse were excluded from the study; those who were unable to cooperate throughout the research process were also excluded to ensure the quality of responses. Patients were enrolled from October 2019 to December 2020 after obtaining written consent and introducing the research center staff. The responses of the self-administered scale and clinical diagnosis of each case were recorded. The study protocol was approved by the Ethical Committee of the First Affiliated Hospital of Chinese Medicine (no. AF/JD-01/05).

### 2.3 Data management and statistical analysis

The records were independently validated and compiled in Excel with the cooperation of two staff. Through the validation of a third staff, data with obvious conflicts or incomplete information were excluded, and the tolerance for the rate of missing data was set as 5%. Reliability was evaluated using the Cronbach’s alpha index and calculated in SPSS 21.0. The assessment framework consisted of five dimensions, including systemic symptoms, upper GI symptoms, lower GI symptoms, environmental sensitivity, and emotional disorders, as shown in Supplementary Table S1. The structural validity of the framework was evaluated via SEM in AMOS 21.0. Metrics such as the goodness-of-fit index (GFI), comparative fit index (CFI), root mean-squared error of approximation (RMSEA), root mean-squared residual (RMR), and Tucker–Lewis index (TLI) were calculated and evaluated. The psychometric properties of each item, including multidimensional discrimination indices (MDISCs) and standardized factor loadings on the latent traits, were estimated using MIRT in R software 3.2.4. The item character and item information curves were also plotted as intuitive representations of the item setting characteristics using the *mirt* R package (Chalmers, 2012).

On the basis of the MIRT model, the CAT scheme was developed with the following settings: (i) initialization: the initial value of each dimension was set as 0 and the range was defined as (−3, 3) before starting the assessment; (ii) scoring: a maximum *a posteriori* (MAP) algorithm was used to estimate the factor score with quasi-Monte-Carlo integration; (iii) pointwise Kullback–Leibler criteria were set as the rule for item selection (Chalmers, 2020); (iv) stopping: using a simulated procedure, the test was terminated when all items were answered or the Standard Error of all latent scores were less than 0.3; (v) discretization: upon estimating the latent traits as continuous indices, equal-width discretization was applied for 3-category division of intensity. The CAT procedures were implemented in a simulation framework using the complete response vectors of all 26 items for each patient. The MAP estimation algorithm was applied to estimate the latent trait scores for all five dimensions. Since all item responses were available for every patient, the stopping rule did not result in data loss or premature termination.

Latent profile analysis (LPA) was used to estimate the sample subgroups based on the latent trait scores from CAT using R 3.2.4 (*tidyLPA* package) by setting Mplus 7.4 as the backend for model fitting. The LCA was also performed on the basis of the original item responses as a comparison. Gaussian mixture modeling (Rasmussen, 1999) was used as the algorithm for estimating models with different numbers of profiles. The following GFIs were estimated and compared among different models: log-likelihood (Loglik), Akaike information criterion (AIC), Bayesian information criterion (BIC), entropy, bootstrap likelihood ratio test (BLRT), and Lo–Mendell–Rubin likelihood ratio test (LMRT). Finally, the model with the most adequate fitness indices was selected for further analysis. Comparisons between IBS and FD based on the demographic descriptors were obtained using Pearson’s Chi-square analysis or ANOVA in R 3.2.4. The Mann–Whitney U test was used to compare the original responses between the subgroups. Moreover, the symptom intensities and estimated latent trait scores were compared using the Kruskal–Wallis H test followed by Nemenyi’s *post hoc* test for pairwise comparisons of the subgroups.

## 3 Results

### 3.1 Participants

A total of 1,124 patients were originally recruited for the research, among which 996 cases were finally enrolled after meeting the inclusion criteria. Of these, 128 cases were excluded during the quality control process. As shown in Table 1, the study group consisted of 487 IBS patients and 509 FD patients according to their first diagnosis, among which females comprised a larger proportion of 53.41% than males. However, this difference in the distribution of sex was evaluated to be not statistically significant based on Pearson’s Chi-squared test (*p* = 0.935). The average age of the group was 38.47 years (range: 18–74 years). Among the enrolled participants, 425 people suffered from discomforts for 1–3 years, accounting for 42.67% of the sample population.

**TABLE 1:** Demographics of the sample population with functional dyspepsia (FD) and irritable bowel syndrome (IBS).

Patient cohort		
Description	Number of participants	996
	Number of female/male participants	532/464
	Percentage of female participants	53.41%
	Age (years)	38.47 (range: 18–74)
Diagnosis	IBS	487 (48.98%)
	FD	509 (51.10%)
Duration (since first diagnosis)	0–1 year	347 (34.84%)
	1–3 years	425 (42.67%)
	More than 3 years	224 (22.49%)

The clinical features between FD and IBS were compared, and these results are shown in Table 2. After applying Benjamini–Hochberg correction, significant differences were observed conspicuously among the typical symptoms for the diagnosis of FD and IBS, including bloating, belching, nausea, diarrhea, lower abdominal pain, loose stools, hyperactive bowel sounds, urgency, and discomfort aggravated by irregular diet, with an adjusted *p* < 0.05. Notably, back pain, joint pain, and heartburn, which showed nominal significance in the unadjusted Mann–Whitney U test (*p* < 0.05), were not significant after adjustment. The other symptoms and influencing factors were evaluated to be not significantly different between the two groups of patients. Anxiety, fatigue, insomnia, and irritation were the most prevalent non-GI symptoms, with each condition being reported by over 50% of patients in both the FD and IBS subgroups.

**TABLE 2:** Comparison of intensity of each symptom between FD and IBS.

Item	Category	Subtypes of functional gastrointestinal disorders (FGIDs)		Z-score	*p*-value	adjusted *p*-value
		FD (n = 509)	IBS (n = 487)			
Back pain	0	439	442	−2.26	<0.05	0.062
	1	63	43			
	2	5	2			
	3	2	0			
Chest pain	0	458	450	−1.32	0.19	0.32
	1	51	35			
	2	0	2			
Joint pain	0	431	387	2.15	<0.05	0.07
	1	74	94			
	2	4	4			
	3	0	2			
Palpitations	0	372	348	0.51	0.61	0.69
	1	125	130			
	2	12	9			
Insomnia	0	219	224	−0.86	0.39	0.56
	1	253	228			
	2	36	34			
	3	1	1			
Fatigue	0	175	168	−1.64	0.11	0.19
	1	211	242			
	2	122	76			
	3	1	1			
Bloating	0	237	316	−6.56	<0.01	<0.01
	1	194	150			
	2	73	17			
	3	5	4			
Abdominal cold	0	428	407	0.38	0.71	0.73
	1	66	53			
	2	9	21			
	3	6	6			
Heartburn	0	417	422	−2.03	<0.05	0.09
	1	80	56			
	2	12	9			
Belching	0	192	283	−7.17	<0.01	<0.01
	1	185	148			
	2	108	48			
	3	24	8			
Nausea	0	425	375	2.55	<0.05	<0.05
	1	72	97			
	2	9	15			
	3	3	0			
Upper abdominal pain	0	401	372	0.9	0.37	0.56
	1	100	107			
	2	8	8			
Early fullness	0	327	333	−1.72	0.08	0.17
	1	104	102			
	2	67	48			
	3	11	4			
Diarrhea	0	392	75	19.6	<0.01	<0.01
	1	113	343			
	2	2	61			
	3	2	8			
Lower abdominal pain	0	412	207	12.5	<0.01	<0.01
	1	97	280			
Loose stools	0	346	121	14.1	<0.01	<0.01
	1	156	303			
	2	7	49			
	3	0	14			
Hyperactive bowel sounds	0	483	361	9.17	<0.01	<0.01
	1	26	110			
	2	0	14			
	3	0	2			
Urgency	0	430	216	180.29	<0.01	<0.01
	1	70	204			
	2	5	37			
	3	4	30			
Irregular diet trigger	0	327	372	−4.16	<0.01	<0.01
	1	182	114			
	2	0	1			
Overwork trigger	0	340	326	−0.05	0.96	0.96
	1	169	161			
Indigestible food trigger	0	246	247	−0.75	0.45	0.57
	1	263	240			
Excitant food trigger	0	291	296	−1.16	0.25	0.4
	1	218	191			
Depression	0	255	248	−0.68	0.5	0.59
	1	147	153			
	2	104	82			
	3	3	4			
Irritation	0	241	208	0.46	0.65	0.7
	1	144	179			
	2	123	98			
	3	1	2			
Anxiety	0	160	160	−0.79	0.43	0.57
	1	220	215			
	2	127	111			
	3	2	1			
Stress	0	242	243	−0.74	0.46	0.57
	1	267	244			

Comparisons of the severity of symptoms between patients with FD and IBS using the Mann–Whitney U test and Benjamini–Hochberg correction.

### 3.2 Validity and reliability

The validity and reliability of the self-administered questionnaire were evaluated according to the assessment framework shown in Supplementary Table S1. The Cronbach’s alpha coefficient of all the items in the instrument was evaluated to be 0.72, and the split-half reliability was calculated as 0.84. The construct validity of the instrument was evaluated via SEM to be adequate, with Chi-square/df = 3.45, CFI = 0.92, GFI = 0.96, RMSEA = 0.05, TLI = 0.90, and RMR = 0.02. The psychometric properties, including MDISCs and factor loading indices, of each item evaluated using MIRT are shown in Supplementary Table S2. All items were evaluated with significant loading on the related factors. Fatigue and insomnia had the highest loading and MDISCs on the systemic symptoms. Similarly, discrimination among bloating, nausea, abdominal cold, heartburn, and early fullness as typical upper GI discomforts were evaluated to be adequate, while belching and upper abdominal pain were evaluated at the lower GI level. Three symptoms overlapped for both FD and IBS but showed no significant differences in severity: abdominal cold (*p* = 0.71), upper abdominal pain (*p* = 0.37), and early fullness (*p* = 0.08). All lower GI symptoms were evaluated to be different in terms of proportion and discrimination. As environmental sensitivity factors, discomforts triggered by indigestible food were evaluated with the highest factor loading as these are the most typical discomforts of both FD and IBS. There were no differences among symptoms in the psychological comorbidity factors, among which anxiety and stress had the highest and lowest factor loadings, respectively.

### 3.3 Latent profile determination with original responses

LPA was used to estimate the hidden patterns in the heterogeneous clinical appearances; based on the original responses to the assessment, the 3-class latent model was evaluated to be the most adequate (*p*-LMRT > 0.05) compared to the 4-class model, as shown in Table 3. The intensities of the clinical features of each estimated subgroup are plotted in Figure 1. The subgroups were named according to their intensities in the spectrum of clinical appearances. The first category was defined as high GI severity with comorbidity subgroup (HGIMS), in which higher intensity GI discomforts, systemic symptoms, and environmental sensitivity were evaluated in comparison with other subgroups. These subgroups share similar intensities of systemic symptoms, environmental trigger factors, and emotional disorders except stress, and the other two subgroups could be differentiated on the basis of the location of manifestation of predominant symptoms. Thus, they were named as the upper-GI-discomfort-predominant subgroup (UGIPS) and lower-GI-discomfort-predominant subgroup (LGIPS).

**TABLE 3:** Latent profile modeling results with the original responses.

Class	AIC	BIC	Entropy	n_min	n_max	BLRT_val	BLRT_p	LMRT_val	LMRT_p
1	38,966.85	39,275.78	1	1	—	—	—	—	—
2	37,374.27	37,997.04	0.88	477/47.90%	519/52.10%	−19420.42	<0.01	1716.69	<0.01
3	36,402.54	37,339.16	0.88	224/22.49%	422/42.37%	−18581.24	<0.01	1,139.36	<0.01
4	35,894.10	37,144.55	0.86	207/20.78%	311/31.23%	−18019.99	<0.01	654.41	0.73

AIC, Akaike information criterion; BIC, Bayesian information criterion; BLRT, bootstrap likelihood ratio test; LMRT, Lo–Mendell–Rubin likelihood ratio test.

**FIGURE 1:**
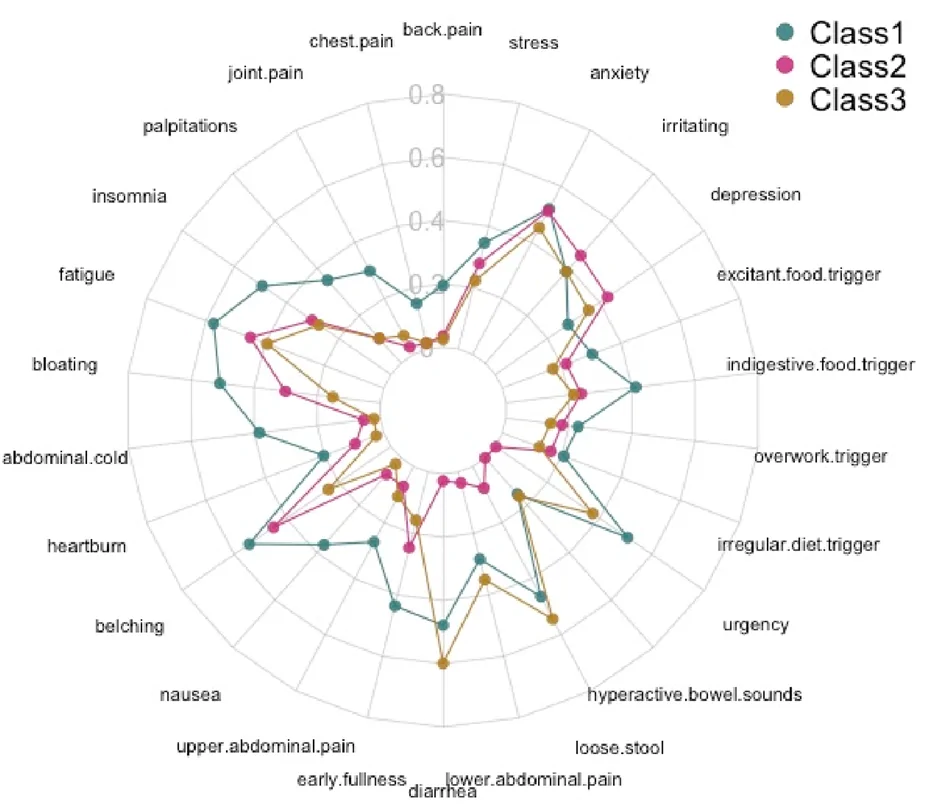
Characteristics of the 3-class latent profile analysis (LPA) model of functional dyspepsia (FD) and irritable bowel syndrome (IBS).

In the UGIPS, moderate intensities of upper GI symptoms showed a statistical difference (*p* < 0.05), including bloating, belching, and early fullness, compared to the other two subgroups (Supplementary Table S3). Moreover, the bowel symptoms, including lower abdominal pain, loose stools, hyperactive bowel sounds, and urgency, were evaluated to have significant lower intensities in the UGIPS. In the LGIPS, higher intensities of diarrhea and lower GI pain alongside lower intensities of symptoms such as bloating, heartburn, belching, early fullness, and urgency showed a significant difference (*p* < 0.05) compared with the UGIPS. Additionally, there was no difference in GI symptoms such as abdominal cold, nausea, and upper abdominal pain between the UGIPS and LGIPS. We also noted no differences in the intensities of irritation for the emotional disorder dimension among the three subgroups. In the HGIMS, FD and IBS accounted for 39.73% and 60.27%, respectively. FD accounted for 84.60% in the UGIPS, while IBS accounted for 82% in the LGIPS. Although differences in the proportions of clinical diagnosis among the subgroups were significantly different (*p* < 0.001) (Supplementary Table S4), the comorbidities of the clinical subtypes are obviously inconsistent between the estimated profiles and the subtypes formally defined using the Rome criteria.

### 3.4 Latent profile modeling with latent trait scores

Using multidimensional computerized adaptive testing (MCAT), we estimated the latent trait score for each patient in the range of −3 to 3. The distribution of these scores is shown in Supplementary Table S5. As presented in Table 4, the 7-profile LPA model was identified as the most adequate candidate, with an entropy of 0.98, *p-*LMRT < 0.01, and *p*-BLRT < 0.01. The 5-profile model met the statistical adequacy criteria and 8-profile model yielded non-significant BLRT and LMRT values (*p* > 0.05), confirming that the 7-profile solution represents the upper bound of statistically justified extraction while capturing all meaningful heterogeneities without overextraction. Importantly, the additional profiles in the 7-profile model are clinically distinct rather than redundant, capturing meaningful differences in the comorbidity patterns and environmental sensitivity that would be obscured in coarser models, with direct implications for treatment prioritization. This granularity enables more precise patient stratification for treatment selection and supports the proposed cascaded interpretation framework that progresses from GI symptom location to comorbidity severity and environmental sensitivity to generate actionable clinical recommendations. As shown in Table 5, the proportions of clinical subtypes (FD vs. IBS) across the seven subgroups were not significantly different (*p* = 0.08). Furthermore, the heterogeneity of latent traits was evident among these subgroups, which are characterized by varying severity levels and combinations of clinical features, as shown in Figure 2.

**TABLE 4:** Latent profile modeling results based on the multidimensional computerized adaptive testing scores.

Class	AIC	BIC	Entropy	n_min	n_max	BLRT_val	BLRT_p	LMRT_val	LMRT_p
1	12,389.65	12,438.68	1.00	1	1	—	—	—	—
2	11,511.61	11,590.07	1.00	492/49.50%	503/50.50%	−6,184.82	<0.01	869.06	<0.01
3	11,404.60	11,512.49	0.91	89/8.94%	493/49.50%	−5,739.80	<0.01	116.20	<0.01
4	11,229.29	11,366.60	0.95	201/20.18%	302/30.32%	−5,680.30	<0.01	182.90	<0.01
5	10,969.98	11,136.71	0.98	48/4.82%	254/25.50%	−5,586.65	<0.01	264.92	<0.01
6	10,926.47	11,122.62	0.95	48/4.82%	252/25.30%	−5,450.99	<0.01	54.20	<0.01
7	10,683.00	10,908.57	0.98	24/2.41%	253/25.40%	−5,328.29	<0.01	64.05	<0.01
8	10,930.92	11,185.91	0.90	5/0.005	236/22.49%	−5,470.95	<0.01	−197.71	1.00

AIC, Akaike information criterion; BIC, Bayesian information criterion; BLRT, bootstrap likelihood ratio test; LMRT, Lo–Mendell–Rubin likelihood ratio test.

**TABLE 5:** Comparison of compositions of the subgroups between FD and IBS for the 7-profile latent profile analysis (LPA).

Subgroup ID	n	Proportion	FD	IBS	Chi-square	*p*-value
1	192	19.28%	92	100	11.391	0.08
2	228	22.89%	117	111		
3	202	20.28%	97	105		
4	49	4.92%	30	19		
5	48	4.82%	30	18		
6	253	25.40%	136	117		
7	24	2.41%	7	17		

**FIGURE 2:**
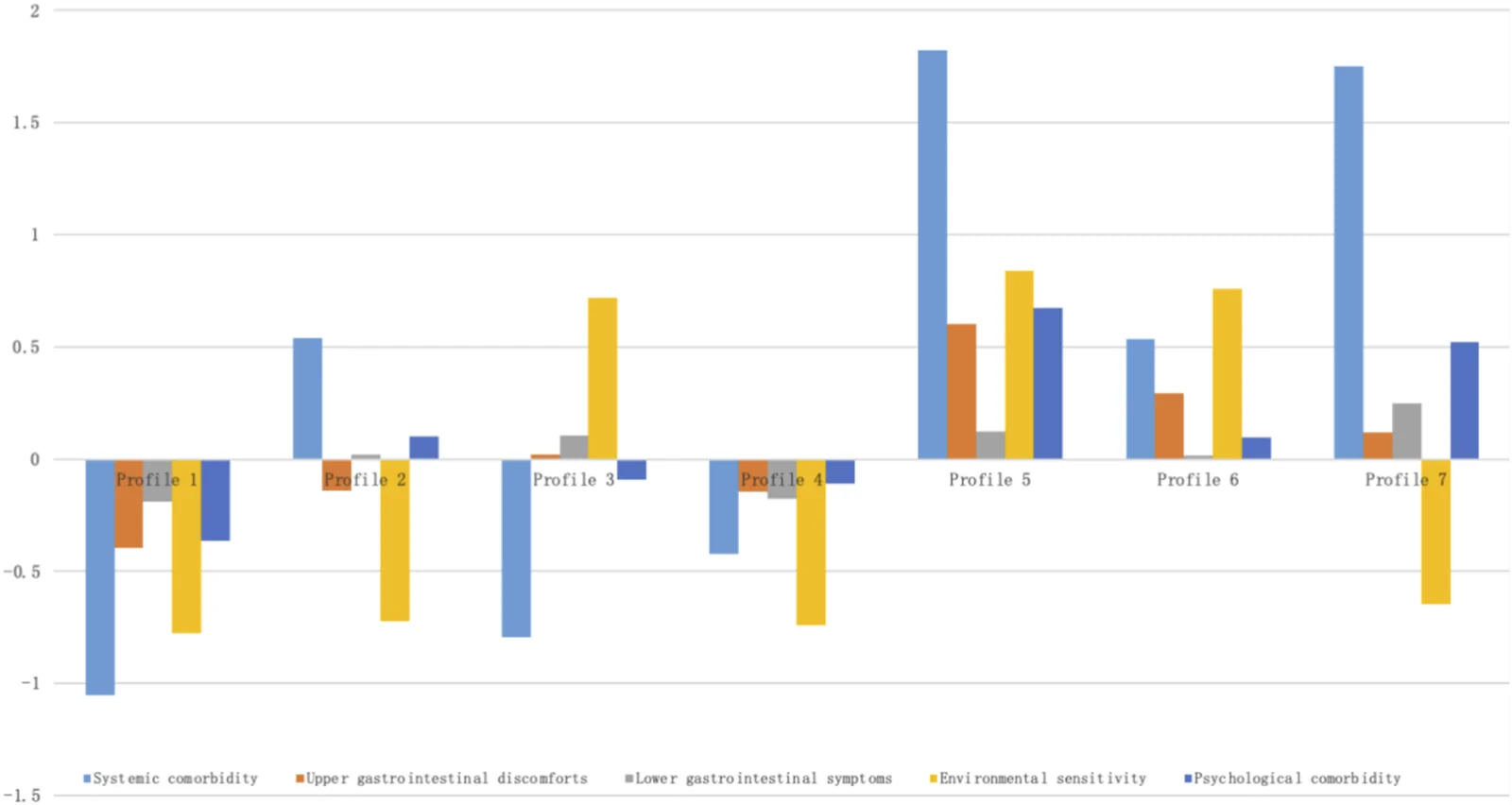
Latent traits scores of the 7-profile LPA model of FD and IBS.

#### 3.4.1 Subgroups defined with predominant GI discomforts

As shown in Supplementary Table S6, the profiles were designated as P1–P7. Subgroups including P3, P5, and P6 were defined as the GI-discomfort-predominant subgroups (GISs), with a higher score in the GI discomforts dimension compared to the average sample condition. Subgroups containing P1, P2, P4, and P7 were defined as mild-GI-severity subgroups (miGISs), with lower scores of GI symptoms. As the severity of lower GI discomforts of all subgroups except P3 were evaluated to be similar, patients in the GISs were further divided into the UGIPS and whole-GI-discomfort subgroup (wPGIS) based on the latent trait scores for GI discomfort. The P3 subgroup comprised about 20.28% of the cohort with 202 patients and was defined as wPGIS as the patients reported significantly higher severity in both upper and lower GI symptoms compared to the miGISs. P5 and P6 were defined as UGIPS and accounted for a total of 30.22% of the cohort with 301 patients, who had significantly higher scores in the upper GI discomforts dimension.

#### 3.4.2 Subgroups defined with severity of comorbidity

As shown in Supplementary Table S5, the latent trait scores for systemic comorbidity ranged from −1.29 to 2.98 (mean = 0.0). Using equal-width discretization, the severity was categorized as mild [−1.5, 0), moderate [0, 1.5), and severe [1.5, 3.0), as visually depicted in Figure 2. Therefore, by cascading to the predominant GI discomforts, the subgroups were further defined according to the severity of comorbidity with systemic and psychological discomforts. As shown in Supplementary Table S6, P1, P3, and P4 were defined as mild comorbidity subgroups with similar lower scores of systemic symptoms than the other subgroups. P2 and P6 were defined as moderate systemic comorbidity, while P5 and P7 were defined as severe comorbidity subgroups. Psychological comorbidities also served as clues for the subgrouping; here, P5 and P7 were defined as higher psychological comorbidity subgroups with higher scores for psychological comorbidity than the other subgroups, while the remaining subgroups were defined as low psychological comorbidity subgroups.

#### 3.4.3 Subgroups defined with environmental sensitivity

It was found that patients with FGIDs are sensitive to social or environmental effects, such as overwork and dietary influences. Statistically significant differences were observed in the environmental sensitivity scores among the subgroups (Kruskal–Wallis H test, pairwise comparisons with Nemenyi’s *post hoc* test, *p* < 0.05). There were 503 patients in the P3, P5, and P6 subgroups, as shown in Table 5; these were defined as environmentally sensitive subgroups with higher scores in the environmental sensitivity dimension than the other subgroups, as shown in Supplementary Table S6. Comparison among the subgroups revealed that patients in the GISs showed positive environmental sensitivity while patients in the miGISs were statistically less sensitive to environmental effects. Thus, the patients in this study could be divided into seven subgroups based on the comprehensive clinical presentations. Accordingly, a clinical diagnosis approach was proposed in a cascaded logic for subgrouping patients with FD and IBS based on their severity and GI discomfort location, severity of extradigestive tract symptoms, emotional discomforts, and environmental sensitivity.

## 4 Discussion

Although the Rome criteria serves as guidance for categorizing FGIDs, it is still challenging to achieve comprehensive management because of the heterogeneity and variability of clinical appearances of the patients. The Rome criteria show limitations in extracting individual symptom complexes, especially when psychology–gastroenterology interactions are garnering more attention in clinical practice. In this study, no difference was found between FD and IBS in emotional disorder symptoms, such as anxiety and irritation. The findings on symptom overlaps between FD and IBS in this study are consistent with those reported in previous clinical studies (Von Wulffen et al., 2019; Pohl et al., 2018). Comparing the subtyping of the Rome criteria, poor concordance was evaluated for the 3-class LPA model covering comprehensive GI symptoms and comorbidities with systemic symptoms and emotional disorders. The comorbidities also indicated similar pathological mechanisms among the different subtypes of FGIDs.

Disorder of the gut–brain interaction plays a bidirectionally crucial role in the pathophysiologies of both FD and IBS (Koloski et al., 2016). Using magnetic resonance imaging and positron emission tomography, researchers have found that there are regional alterations to the central nervous system in these two subtypes (Larsson et al., 2012; Van Oudenhove et al., 2010). Previous studies have shown that the homeostases of mucosal cytokines and gut-homing T lymphocytes were disrupted in both FD and IBS (Burns et al., 2019; McCullough, 2013). It was also proposed that the comorbidities could be associated with hypersensitivity, motility abnormalities in the GI tract, and functional alterations of the hypothalamic-pituitary-adrenal axis and the sympathetic nervous system (Lackner, 2014). Therefore, attention should be focused on the prevalence of comprehensive clinical features in individuals and systemic clues to the pathogenesis of FGIDs (Holtmann and Talley, 2018).

The results of the LPA show that patients with different subtypes of FGIDs could be clustered into subgroups based on similarities in their symptom combination and severity, which were evaluated to be statistically significant and distinguishable among the subgroups. In the 3-class LPA model estimated with the original responses for the intensity of symptoms, the HGIMS included similar proportions of the FD and IBS subtypes. Unlike the categorization based on rules in the Rome criteria, both the intensity of GI symptoms and comorbidities with psychological and systemic symptoms serve as clues for defining the subgroups. This means that the heterogeneity of the FGIDs can be captured by considering not only the intensity but also combination of symptoms. The patterns identified by the model also reiterate that it is essential to utilize all clinical feature occurrences to explore the pathogenesis of FGIDs.

Many instruments have been developed in the form of scales for subgrouping FGIDs (Bouchoucha et al., 2020; Zinsmeister et al., 2018). However, the compensatory approach via accumulating response scores overlays details about the individuals and diminishes the interpretation of the assessment. Therefore, we quantified the latent traits as a summarized mixture of symptoms based on the MIRT model in an uncompensatory algorithm. Using the MCAT results estimated from the 5-dimensional model, the latent traits of each patient were evaluated for upper and lower GI discomforts, severity of emotional disorders, severity of systemic symptoms, and environmental sensitivity. As continuous variables, the latent trait scores benefit the assessment with more comparable results and better interpretability than traditional calculation methods.

The LPA model based on the MCAT approach indicates that the enrolled patients with FD and IBS could be divided into seven subgroups on basis of five latent trait scores. Thus, a cascaded interpretation of the model was proposed by considering the severity of GI symptoms, severity of comorbidities, and sensitivity to environmental factors. The differences among these subgroups represent the heterogeneity of the FGIDs and also remind us of the importance of individualized treatments for patients with FGIDs according to their comprehensive clinical appearances. Since the sample size was limited in this study, the stability of the subgrouping results should be further validated with more representative samples. Furthermore, more objective evidence is needed to explore the pathological differences among the subgroups and promote the clinical application of the model.

To the best of our knowledge, this is the first study in China that proposes a systematic paradigm for patient-centered subgrouping of FGIDs by applying MCAT and LPA. Based on an uncompensatory algorithm for estimating individual latent traits, loss of information was eliminated during MCAT by displacing the traditional setting of the assessment in which accumulative rules are commonly applied. By taking personal traits other than symptom clusters as parameters, the model interpretation was strengthened for clinical practice. However, there are several limitations to our research. First, the current study employs a cross-sectional design. The 7-subtype classification was derived from latent trait profiles estimated at a single timepoint and therefore represents a snapshot of the dynamic disease process rather than a fixed diagnostic taxonomy. FGIDs are characterized by symptom fluctuations over time, with alternating periods of exacerbation and remission. The subtypes identified herein should therefore be interpreted as stage-specific phenotypic snapshots rather than stable immutable categories. Longitudinal studies with repeated measurements are warranted to validate subtype stability, observe potential transitions between the subtypes, and identify predictors of disease trajectory changes. Second, all patients were recruited from a single research center at a tertiary hospital, which could introduce referral bias and limit the representativeness of the sample. Patients seeking care at tertiary institutions may represent a more severely affected subset of the FGID population. The proposed instrument and 7-subtype classification should therefore be revalidated in multicenter studies with more representative samples, including primary care and community-based populations, to establish external validity and generalizability. Third, although the self-administered questionnaire was developed through a strict procedure, the item pool and its psychometric properties should be further optimized and revalidated in independent samples. Such refinement would reduce the bias introduced by unrepresentative sampling and improve the interpretability and extrapolation of the latent profile models. Fourth, the current form of the electronic assessment requires practitioner guidance during administration, which may limit its scalability for independent patient use. Misunderstanding of the item content or test procedures could compromise response validity and patient compliance. Future developments should focus on more intelligent interaction design, including built-in instructions, real-time clarification prompts, progress monitoring, and automated quality checks, to enable safer and more valid self-administration in routine clinical practice. Finally, the assessment logic of the MCAT method could be further optimized by analyzing and comparing the time cost and measurement consistency across different CAT stopping rules and item selection algorithms.

## 5 Conclusion

Considering the comprehensive clinical features of patients with FGIDs, our study proposes a new subgrouping model for FD and IBS using MCAT and LPA. The mixed manifestations in FD and IBS patients could be profiled into seven subgroups based on the severity and combination of symptoms, including GI symptoms, systemic symptoms, and environmental factors. The results suggest that the heterogeneity of patients with FGIDs should be considered to optimize the treatment outcomes. Furthermore, the proposed interdisciplinary strategy is expected to benefit clinicians with approaches for comprehensive evaluation and management of patients; this can shift the clinical practices to an individualized mode through additional flexibility and availability.

## Data Availability

The raw data supporting the conclusions of this article will be made available by the authors without undue reservation.
