# Supplementary material for: Exploring the psychogastroenterology patterns of functional dyspepsia and irritable bowel syndrome comorbidities based on latent profile analysis
Source: Front Pharmacol. 2026 Sep 16;17:1759170. doi: 10.3389/fphar.2026.1759170 (PMC13624401; doi:10.3389/fphar.2026.1759170)
Supplement: Supplementary file 1 [file Supplementaryfile1.docx]

**Table S1 Framework of the assessment for FGIDs with 5 latent traits**

| **Index** | **Latent traits** | **Symptoms or factors for assessment** |
| --- | --- | --- |
| 1 | Systemic symptoms | Back Pain; Chest Pain; Joint Pain; Palpitations; Insomnia; Fatigue |
| 2 | Upper gastrointestinal symptoms | Bloating; Abdominal Cold; Heartburn; Belching; Nausea; Upper Abdominal Pain; Early Fullness |
| 3 | Lower gastrointestinal symptoms | Diarrhea; Lower Abdominal Pain; Loose Stool; Hyperactive Bowel Sounds; Urgency |
| 4 | Environmental sensitivity | Irregular Diet Trigger; Overwork Trigger; Indigestive Food Trigger; Excitant Food Trigger |
| 5 | Emotional disorders | Depression; Irritating; Anxiety; Stress |

**Table S2 Psychometric properties of each item of the self-administrated questionnaire**

| **Item** | **MDISC** | **Factor loadings** | | | | |
| --- | --- | --- | --- | --- | --- | --- |
|  |  | **Systemic comorbidity** | **Upper gastrointestinal discomforts** | **Lower gastrointestinal symptoms** | **Environmental sensitivity** | **Psychological comorbidity** |
| back pain | 0.47 | 0.27 | 0.00 | 0.00 | 0.00 | 0.00 |
| chest pain | 0.52 | 0.29 | 0.00 | 0.00 | 0.00 | 0.00 |
| joint pain | 0.42 | 0.24 | 0.00 | 0.00 | 0.00 | 0.00 |
| palpitations | 0.480 | 0.27 | 0.00 | 0.00 | 0.00 | 0.00 |
| insomnia | 2.96 | 0.99 | 0.00 | 0.00 | 0.00 | 0.00 |
| fatigue | 2.95 | 0.87 | 0.00 | 0.00 | 0.00 | 0.00 |
| bloating | 1.54 | 0.00 | 0.67 | 0.00 | 0.00 | 0.00 |
| abdominal cold | 1.50 | 0.00 | 0.66 | 0.00 | 0.00 | 0.00 |
| heartburn | 1.18 | 0.00 | 0.57 | 0.00 | 0.00 | 0.00 |
| belching | 0.73 | 0.00 | 0.39 | 0.00 | 0.00 | 0.00 |
| nausea | 1.66 | 0.00 | 0.70 | 0.00 | 0.00 | 0.00 |
| upper abdominal pain | 0.79 | 0.00 | 0.42 | 0.00 | 0.00 | 0.00 |
| early fullness | 1.14 | 0.00 | 0.56 | 0.00 | 0.00 | 0.00 |
| diarrhea | 4.11 | 0.00 | 0.00 | 0.92 | 0.00 | 0.00 |
| lower abdominal pain | 1.45 | 0.00 | 0.00 | 0.65 | 0.00 | 0.00 |
| loose stool | 2.55 | 0.00 | 0.00 | 0.83 | 0.00 | 0.00 |
| hyperactive bowel sounds | 2.53 | 0.00 | 0.00 | 0.83 | 0.00 | 0.00 |
| urgency | 2.31 | 0.00 | 0.00 | 0.81 | 0.00 | 0.00 |
| irregular diet trigger | 0.70 | 0.00 | 0.00 | 0.00 | 0.38 | 0.00 |
| overwork trigger | 1.01 | 0.00 | 0.00 | 0.00 | 0.51 | 0.00 |
| indigestive food trigger | 4.74 | 0.00 | 0.00 | 0.00 | 0.94 | 0.00 |
| excitant food trigger | 0.95 | 0.00 | 0.00 | 0.00 | 0.49 | 0.00 |
| depression | 2.23 | 0.00 | 0.00 | 0.00 | 0.00 | 0.79 |
| irritating | 1.94 | 0.00 | 0.00 | 0.00 | 0.00 | 0.75 |
| anxiety | 4.08 | 0.00 | 0.00 | 0.00 | 0.00 | 0.92 |
| stress | 0.65 | 0.00 | 0.00 | 0.00 | 0.00 | 0.36 |

**Table S3 Comparison of symptom severity among the 3-class subgroups estimated with latent profile analysis**

| **Item content** |  | **Comparison between subgroups** | | | |
| --- | --- | --- | --- | --- | --- |
|  | **Chi-square** | **df** | **LGIPS-HGIMS** | **LGIPS- UGIPS** | **UGIPS-HGIMS** |
| back pain | 126.35^**^ | 2 | 131.96^**^ | 8.11 | 140.07^**^ |
| chest pain | 115.78^**^ | 2 | 115.85^**^ | 0.79 | 115.06^**^ |
| joint pain | 219.38^**^ | 2 | 228.14^**^ | 37.44 | 58.93^**^ |
| palpitations | 139.33^**^ | 2 | 201.98^**^ | 3.30 | 198.68^**^ |
| insomnia | 60.48^**^ | 2 | 142.11^**^ | 18.52 | 160.64^**^ |
| fatigue | 32.28^**^ | 2 | 92.43^**^ | 49.79 | 58.93^**^ |
| bloating | 141.77^**^ | 2 | 162.06^**^ | 97.88^**^ | 259.94^**^ |
| abdominal cold | 292.39^**^ | 2 | 228.07^**^ | 21.26 | 249.33^**^ |
| heartburn | 80.25^**^ | 2 | 84.00^**^ | 55.39^**^ | 139.40^**^ |
| belching | 79.85^**^ | 2 | 73.35^**^ | 120.45^**^ | 193.80^**^ |
| nausea | 303.4^**^ | 2 | 247.74^**^ | 28.91 | 276.65^**^ |
| upper abdominal pain | 102.33^**^ | 2 | 171.44^**^ | 35.30 | 136.14^**^ |
| early fullness | 82.801^**^ | 2 | 134.19^**^ | 50.84^**^ | 185.03^**^ |
| diarrhea | 682.84^**^ | 2 | 364.01^**^ | 463.71^**^ | 99.69^**^ |
| lower abdominal pain | 312.21^**^ | 2 | 231.52^**^ | 294.21^**^ | 62.69^**^ |
| loose stool | 461.78^**^ | 2 | 331.13^**^ | 367.40^**^ | 36.28 |
| hyperactive bowel sounds | 131.62^**^ | 2 | 136.29^**^ | 129.05^**^ | 7.25 |
| urgency | 391.12^**^ | 2 | 351.71^**^ | 266.70^**^ | 85.01^**^ |
| irregular diet trigger | 16.03^**^ | 2 | 41.15 | 36.52 | 77.67^**^ |
| overwork trigger | 16.36^**^ | 2 | 47.04 | 34.07 | 81.10^**^ |
| indigestive food trigger | 84.07^**^ | 2 | 161.58^**^ | 22.42 | 183.99^**^ |
| excitant food trigger | 34.57^**^ | 2 | 81.73^**^ | 41.26 | 122.99^**^ |
| depression | 14.92^**^ | 2 | 80.93^**^ | 46.24 | 34.69 |
| irritating | 4.38 | 2 | 27.85 | 39.31 | 11.45 |
| anxiety | 6.39^*^ | 2 | 12.64 | 39.47 | 52.11 |
| stress | 29.14^**^ | 2 | 62.75^**^ | 51.88^**^ | 114.63^**^ |

Comparing severity of symptoms of subgroups using Kruskal-Wallis H test. **:p<0.01,*:p<0.05; HGIMS: high GI severity with comorbidity subgroup; UGIPS: upper GI discomfort-predominate subgroup; LGIPS: lower GI discomfort-predominate subgroup.

**Table S4 Comparison of proportion for the 3-class LPA subgroups between FD and IBS**

| **Group ID** | **n** | **Proportion** | **FD** | **IBS** | **Chi-square** | **P value** |
| --- | --- | --- | --- | --- | --- | --- |
| Class 1 | 224 | 22.49% | 89 | 135 | 354.54 | <0.01 |
| Class 2 | 422 | 42.37% | 357 | 65 |  |  |
| Class 3 | 350 | 35.14% | 63 | 287 |  |  |

**Table S5 Discretization of latent traits estimated with multidimensional computerized adaptive testing**

| **Latent trait** | **Minimum** | **Mean** | **Maximum** |
| --- | --- | --- | --- |
| Systemic comorbidity | -1.29 | 0.00 | 2.98 |
| Upper gastrointestinal discomforts | -1.15 | 0.00 | 2.07 |
| Lower gastrointestinal symptoms | -1.19 | 0.00 | 2.65 |
| Environmental sensitivity | -1.12 | 0.01 | 1.92 |
| Psychological comorbidity | -1.38 | 0.00 | 2.99 |

**Table S6 Comparison of latent traits severity among the 7-profile LPA subgroups**

| **Factor** | **Comparison between subgroups** | | | | | | | | | | | | | | | | | | | | | | |
| --- | --- | --- | --- | --- | --- | --- | --- | --- | --- | --- | --- | --- | --- | --- | --- | --- | --- | --- | --- | --- | --- | --- | --- |
|  | **Chi-square(p value)** | **df** | **P1-P2** | **P1-P3** | **P1-P4** | **P1-P5** | **P1-P6** | **P1-P7** | **P2-P3** | **P2-P4** | **P2-P5** | **P2-P6** | **P2-P7** | **P3-P4** | **P3-P5** | **P3-P6** | **P3-P7** | **P4-P5** | **P4-P6** | **P4-P7** | **P5-P6** | **P5-P7** | **P6-P7** |
| Systemic comorbidity | 821.98^**^ | 6 | 526.52^**^ | 99.58 | 220.00^**^ | 808.91^**^ | 536.44^**^ | 806.91^**^ | 426.95 | 306.52^**^ | 282.38^**^ | 9.91 | 280.38^**^ | 120.43 | 709.33^**^ | 436.86^**^ | 707.33^**^ | 588.90^**^ | 316.43^**^ | 586.90^**^ | 272.47^**^ | 2.00 | 270.47^**^ |
| Upper gastrointestinal discomforts | 124.82^**^ | 6 | 94.04 | 157.14^**^ | 105.54 | 371.96^**^ | 254.31^**^ | 209.67 | 63.10 | 11.50 | 277.91^**^ | 160.26^**^ | 115.62 | 51.60 | 214.82^**^ | 97.17^*^ | 52.52 | 266.41 | 148.76 | 104.12 | 117.65 | 162.29 | 44.64 |
| Lower gastrointestinal symptoms | 17.678^**^ | 6 | 72.77 | 105.23^*^ | 38.86 | 97.49 | 72.37 | 159.97 | 32.46 | 33.91 | 24.72 | 0.40 | 87.20 | 66.37 | 7.74 | 32.86 | 54.74 | 58.64 | 33.51 | 121.11 | 25.12 | 62.48 | 87.60 |
| Environmental sensitivity | 749.25^**^ | 6 | 21.73 | 500.47^**^ | 16.47 | 553.31^**^ | 516.09^**^ | 75.62 | 478.73^**^ | 5.26 | 531.57^**^ | 494.36^**^ | 53.89 | 484.00^**^ | 52.84 | 15.62 | 424.85^**^ | 536.84^**^ | 499.62^**^ | 59.15 | 37.21 | 477.69^**^ | 440.47^**^ |
| Psychological comorbidity | 79.327^**^ | 6 | 151.39^**^ | 96.41 | 81.50 | 341.80^**^ | 157.18^**^ | 288.48^**^ | 54.98 | 69.90 | 190.40^**^ | 5.79 | 137.09 | 14.91 | 245.39^**^ | 60.77 | 192.08 | 260.30^**^ | 75.68 | 206.99 | 184.62^*^ | 53.31 | 131.31 |

Comparing severity of symptoms of subgroups using Kruskal-Wallis H test. P1-P7:Profile 1-Profile 7, **:p<0.01,*:p<0.05.
